# Stability and conformational memory of electrosprayed and rehydrated bacteriophage MS2 virus coat proteins

**DOI:** 10.1016/j.crstbi.2022.10.001

**Published:** 2022-11-04

**Authors:** Maxim N. Brodmerkel, Emiliano De Santis, Charlotte Uetrecht, Carl Caleman, Erik G. Marklund

**Affiliations:** a Department of Chemistry - BMC, Uppsala University, Box 576, Uppsala, 75123, Sweden; b Department of Physics and Astronomy, Uppsala University, Box 516, Uppsala, 75120, Sweden; c Leibniz Institute of Virology (LIV), Hamburg, 20251, Germany; d Centre for Structural Systems Biology (CSSB), Deutsches Elektronen-Synchrotron, DESY, Notkestrasse 85, Hamburg, 22607, Germany; e School of Life Sciences, University of Siegen, Siegen, Germany; f Center for Free-Electron Laser Science, DESY, Notkestrasse 85, Hamburg, 22607, Germany

**Keywords:** Molecular dynamics simulations, Bacteriophage, Gas-phase structure, Protein structure, Solvation, Electrospray ionization

## Abstract

Proteins are innately dynamic, which is important for their functions, but which also poses significant challenges when studying their structures. Gas-phase techniques can utilise separation and a range of sample manipulations to transcend some of the limitations of conventional techniques for structural biology in crystalline or solution phase, and isolate different states for separate interrogation. However, the transfer from solution to the gas phase risks affecting the structures, and it is unclear to what extent different conformations remain distinct in the gas phase, and if resolution *in silico* can recover the native conformations and their differences. Here, we use extensive molecular dynamics simulations to study the two distinct conformations of dimeric capsid protein of the MS2 bacteriophage. The protein undergoes notable restructuring of its peripheral parts in the gas phase, but subsequent simulation in solvent largely recovers the native structure. Our results suggest that despite some structural loss due to the experimental conditions, gas-phase structural biology techniques provide meaningful data that inform not only about the structures but also conformational dynamics of proteins.

## Highlights

• Presented extensive molecular dynamics (MD) simulation data investigating protein vacuum exposure and rehydration dynamics**.**
• Demonstrated that the majority of the protein structure recovers their initial solution conformation after vacuum exposure.
• Explored the potential gain for structural biology of using MD simulation to refine gas-phase determined protein structures.

## Graphical abstract

**Figure:**
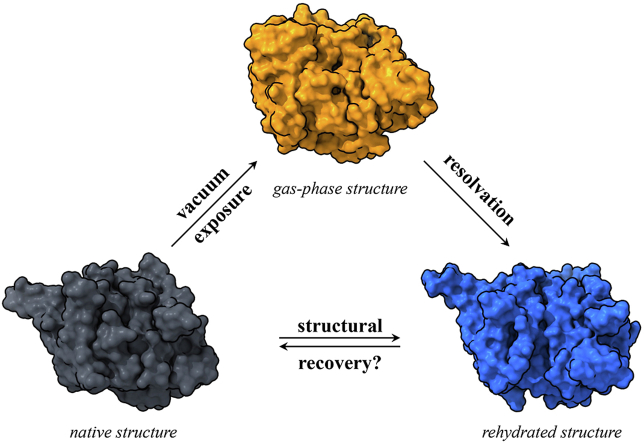

## 1 Introduction

Protein structure determination and investigation are key steps in understanding the function and dysfunction of proteins (Marcoux and Robinson, 2013). A vast range of methods, such as X-ray crystallography and nuclear magnetic resonance spectroscopy, have long been established as dominating methods in structural biology, albeit having limits in their application (Kaur et al., 2018). Mass spectrometry techniques on the other hand allow the investigation of a wider size range and composition of samples, which present challenges for other methods. In combination with ion mobility mass spectrometry, native mass spectrometry is increasingly used to probe the structures and architectures of selected protein states in the gas phase, and in conjunction with single particle imaging it can in principle produce atomistic gas-phase structures (Uetrecht et al., 2011; Morton et al., 2008; Duelfer et al., 2019; Kadek et al., 2021). Native mass spectrometry and related techniques can also be used to mass- and size select proteins and complexes as a preparatory step for subsequent interrogation of structures using other techniques. Notably, this approach has been used to deposit proteins on surfaces for transmission electron microscopy and atomic force microscopy (Benesch et al., 2010; Mikhailov et al., 2014), scanning tunneling microscopy (Deng et al., 2012), electron holography (Longchamp et al., 2017), and recently cryo-EM (Westphall et al., 2022; Esser et al., 2022a,b). As such, there is an emerging range of diverse techniques for structural biology that can determine the structures of proteins during or after time spent in the gas phase. However, transferring proteins from their native environment in solution into vacuum can potentially affect their structures (Patriksson et al., 2007; Rolland and Prell, 2019; Bakhtiari and Konermann, 2019; Breuker and McLafferty, 2008). The vacuum exposure *per se* constitutes a drastically different environment for the proteins; deliberate or unwanted activation can cause unfolding or rearrangement; and the commonly used electrospray ionization (ESI), albeit being a “gentle” ionization technique, is a process that can impact the balance of forces holding a structure together (Abramsson et al., 2021; Sahin et al., 2021). As a result, protein models provided by structural determination techniques of gas-phase complexes might diverge from the true native conformations in solution, and some proteins are indeed challenging to transfer to the gas phase without disrupting their structures. Importantly however, numerous examples show that the structural perturbations can in fact often be kept at a low level for folded proteins under the right conditions (Ouyang et al., 2003; Ruotolo et al., 2005; Marklund et al., 2009; Rolland and Prell, 2019; Seo et al., 2016; Wyttenbach and Bowers, 2011; Meyer et al., 2013; Rolland et al., 2022). Early work demonstrated how intact viruses remained infectuous (Siuzdak et al., 1996) and enzymes retained their activity (Ouyang et al., 2003) after ESI, and how large ring-shaped protein complexes kept their quaternary structre in the gas phase (Ruotolo et al., 2005), which has since been corroborated by a substantial number of experiments and computations. Nonetheless, details about conformational changes upon vacuum-exposure are difficult to obtain from conventional methods, but are important for drawing biologically and physically sound conclusions from gas-phase experiments. One potential approach to look into this, and to recover the native structures, is to rehydrate the obtained vacuum structures *in silico* using molecular dynamics (MD) simulations. A first application of that approach is given by Meyer et al., who showed a series of monomeric proteins to largely recover their *in vitro* structure through the rewetting after vacuum exposure (Meyer et al., 2009) using MD simulations. However, pinpointing the exact differences between vacuum and rehydrated structures might yet prove challenging, and requires suitable model systems. We address this question by interrogating dimeric viral capsid proteins that have distinct and co-existing conformations in the caspsid, enabling us to assess not only how the vacuum might change the structure, but also how different naturally occurring starting conformations affect the gas-phase and resolvated structures.

Viruses in general are classified as non-living, infectious agents, which hijack and reprogram the molecular machinery of a host cell for viral reproduction. The main architecture of viruses comprises, in the most simple case, the viral genome (DNA or RNA), encapsulated by a protective protein shell, the capsid (Buzón et al., 2020). Capsids play a major role for the viral replication cycle by protecting and delivering the genetic information of the virus (Perilla et al., 2016). Especially the self-assembly of the viral capsid is an inevitable and essential step, creating more virions able to infect more cells of the same host, potentially transferring and infecting other organisms (Mateu, 2013). As such, investigating capsids, their coat proteins and assembly process has the potential to give valuable insight in the underlying pathways of the viral life cycle, eventually aiding the development of antiviral therapies and drugs (Perilla et al., 2016; Ode et al., 2012; Liu et al., 2018; Bzówka et al., 2020).

**Fig. 1:**
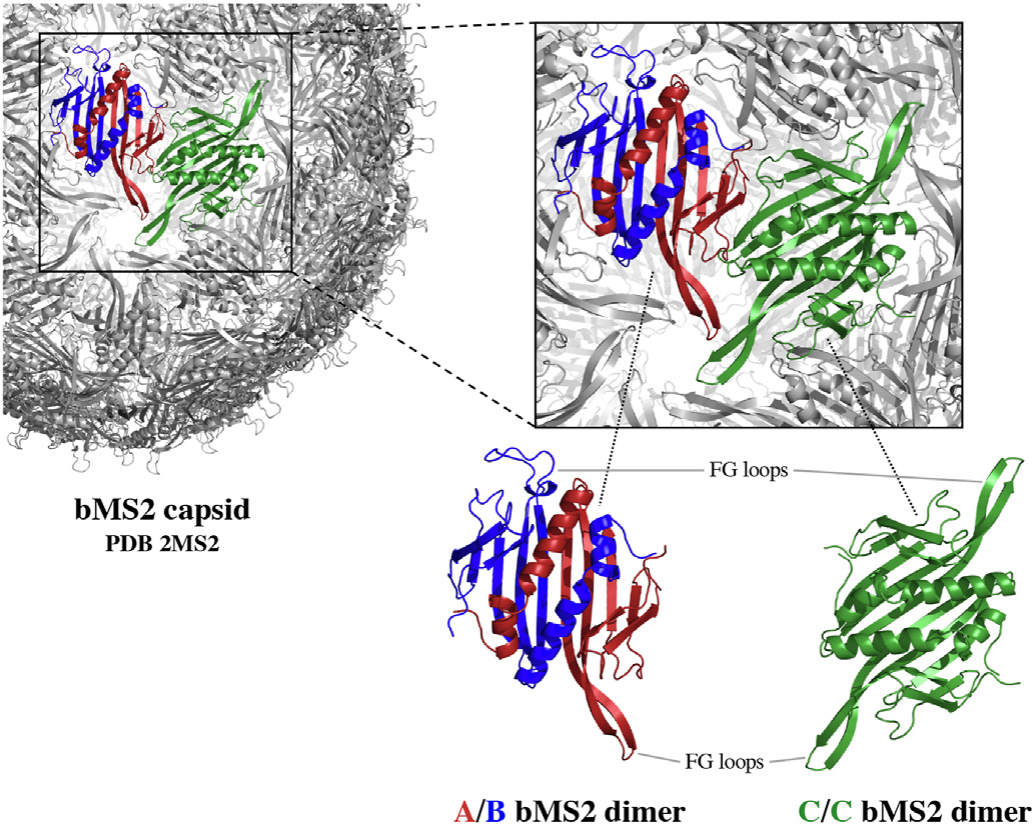
**The bMS2 capsid.** The capsid of the bMS2 virus holds an interesting feature, as it is comprised of two sequence-identical dimers: the asymmetric A/B and symmetric C/C dimer. Predominantly similar in their structure, the main divergence is the well-defined, extended FG loop in **A** and **C** protein chains, which is disordered in **B** chains.

The MS2 bacteriophage (bMS2) is a small RNA-virus of icosahedral symmetry which infects *E. coli* bacteria. The capsid of the native virus consists of 89 dimers of two sequence-identical monomeric protein chains, each comprising 129 amino acids, and one 393 residue long maturation protein, whereas the virus-like particle of the bMS2 virus forms a stable, closed shell consisting of 90 coat protein dimers (Golmohammadi et al., 1993; Dai et al., 2017; Jana and May 2020). On closer look, bMS2 possesses a rather interesting feature. The monomeric capsid proteins are able to adopt three distinctive conformations, commonly indicated as A, B and C, which ultimately leads to the formation of two distinctive dimers: the symmetric C/C dimer, and the asymmetric A/B dimer, classifying the bMS2 capsid as a *T* ​= ​3 structure (Stockley et al., 2007; Valegård et al., 1990). The conformations of both A/B and C/C dimers are predominantly alike, with distinct differences around the FG loop (Golmohammadi et al., 1993), as shown in Fig. 1. Capsid monomers of the A and C conformations have an anti-parallel *β*-hairpin, extended FG loop, whilst those in the B conformation form a disordered, flexible loop between the F and G *β*-strands, where the loop is folded back closer to the protein structure. These structural differences between the A/B and C/C dimers are responsible for the 3-fold and 5-fold symmetry axes of the viral capsid: the 3-fold axes are formed by three interlocking A and C monomers in an alternating pattern, whereas the disordered FG loops of five A/B dimers define the 5-fold axes (Perkett et al., 2016; Stockley et al., 2007). Both coat protein dimers, symmetric and asymmetric, are crucial for an efficient capsid assembly, and thus need to be available for the process to take place properly. A solution purely consisting of coat proteins will predominately feature dimers in the symmetric C/C conformation (Stockley et al., 2007). Upon binding of the 19-nucleotide stem-loop of the viral RNA genome with its binding pocket located on the bottom of a coat protein dimer, the previous symmetric conformation will change into the asymmetric A/B form, therefore providing the necessary parts for a successful capsid assembly (Morton et al., 2010; ElSawy et al., 2010). Consequently, the absence or excess of the genomic RNA results in an insufficent assembly process, yielding only a low number of mature bMS2 capsids.

Identifying crucial steps and dynamics of viral capsids and their proteins prompted the usage of computational resources and methods, where MD simulations have proven particularly useful, especially for computational virology (Brini et al., 2020; Machado and Pantano, 2021; Bruinsma et al., 2021). Complementing experimental data, MD provides detailed, high-resolution information of biomolecules on femto- to microsecond timescales, which are often inaccessible to conventional methods (Marklund and Benesch, 2019). The genome-induced conformational switching from the symmetric C/C dimer into its asymmetric form and its role in the assembly process of the bMS2 capsid has been subject to a manifold of studies, experimentally and theoretically, with MD simulations being increasingly employed to investigate that matter (Perkett et al., 2016; Farafonov and Nerukh, 2019; Lalwani Prakash and Gosavi, 2021; Wang et al., 2019). Simulations of the entire bMS2 capsid gave insight into the distinct transport of water and ions in and out of the capsid shell (Farafonov and Nerukh, 2019), and models of its unique dimers gave valuable insight into the fundamental dynamics of the switching mechanism from a C/C to an A/B conformation (Perkett et al., 2016; Lalwani Prakash and Gosavi, 2021). Moreover, an extensive study was published by Wang et al., investigating the self-assembly process of the bMS2 capsid using a coarse-grained model (Wang et al., 2019). Yet, so far, no study has investigated the stability and dynamics of the bMS2 coat proteins in vacuum, and with it potential conformational changes the structures might experience.

Consequently, the here presented study intends to explore the dynamics of multimeric proteins taken from solution into vacuum: to what extent do the structures respond to the vacuum, and would they find back to their native solution structure by the means of *in silico* rehydration? To this end, we employ MD simulations, which provide the ideal toolset to pinpoint the definitive details of a potential recovery process, to identify structural differences and obtain conformations closer to the native structure (Meyer et al., 2009). We apply our computations to both conformations of the bMS2 capsid dimer protein in extensive simulations in both gas phase and subsequent rehydration, shedding light into their most important dynamics and conformational transitions.

## 2 Methods

### 2.1 Bulk solution MD simulations

In this study, we investigated the dynamics of the bMS2 virus coat protein dimer, in both its C/C and A/B form. The dimer structures were extracted from the Protein Databank entry 2MS2, and refined by adding missing residues using the UCSF Chimera tool (version 1.14) (Pettersen et al., 2004). We employed the Gromacs software package (version 4.5.7) for our simulations (Hess et al., 2008), which were performed on the *Rackham* cluster of the Uppsala Multidisciplinary Center for Advanced Computational Science (UPPMAX). The comparison of the stability of simulated proteins in vacuum using different force fields indicated a minimal influence of the choice of force field on the protein stability (Marklund et al., 2009). Hence, as in our previous studies (Sinelnikova et al., 2021; Marklund et al., 2009, 2017; Patriksson et al., 2007; Mandl et al., 2020), the OPLS-AA force field was utilized to estimate the forces between the atoms (Kaminski et al., 2001) with applied virtual sites (Feenstra et al., 1999). The protein structures were placed under periodic boundary conditions in a dodecahedral box. Subsequently, the structures were solvated using the TIP4P model (Jorgensen et al., 1983) and neutralized by adding a 154 ​mM NaCl solution, where the pKa of the protein side chains at pH 7 determined their protonation state.

The solvated and neutralized systems were minimized using the steepest descent algorithm, and afterwards simulated for 50 ps with applied position-restraints. The temperature was set to 300 ​K, with a coupling constant of *τ* equal to 0.2 ps, using the velocity rescaling thermostat (Bussi et al., 2007). Following temperature coupling, the pressure in the system was maintained at 1 ​bar with a time coupling of 1 ps by employing the Berendsen barostat (Berendsen et al., 1984). Temperature and pressure coupling were applied over 5 ns, respectively. Bonds were constrained by applying the LINCS algorithm (Hess et al., 1997). Afterwards, a simulation of 50 ns, with a 2.5 fs time step, was started, allowing the dimers to relax in solution. Coulomb interactions were calculated with the particle mesh Ewald algorithm at a real-space cut-off of 1 ​nm (Parrinello and Rahman, 1981). Of the last 40 ns of the relax simulation, frames were extracted every 4 ns as starting structures for a total of 10 bulk simulations. Eventually, starting from these relax simulations structures, the systems were simulated with a time step of 4 fs for a total of 750 ns in solution. The goal of the solution simulations was to provide starting structures for vacuum simulations. We calculated the root mean square deviation (RMSD) to estimate how the proteins adjusted to the solvent, and to provide information about the behaviour of the system. We found that the last 160 ns of the bulk simulations showed a near-constant RMSD, and thus we extracted a total of 20 frames without solvent and ions, one every 8 ns, as starting structures of the vacuum simulations. Please see the Supplementary Information (SI), *Data analysis protocol*, for more information regarding data analysis.

### 2.2 Vacuum MD simulations

**Fig. 2:**
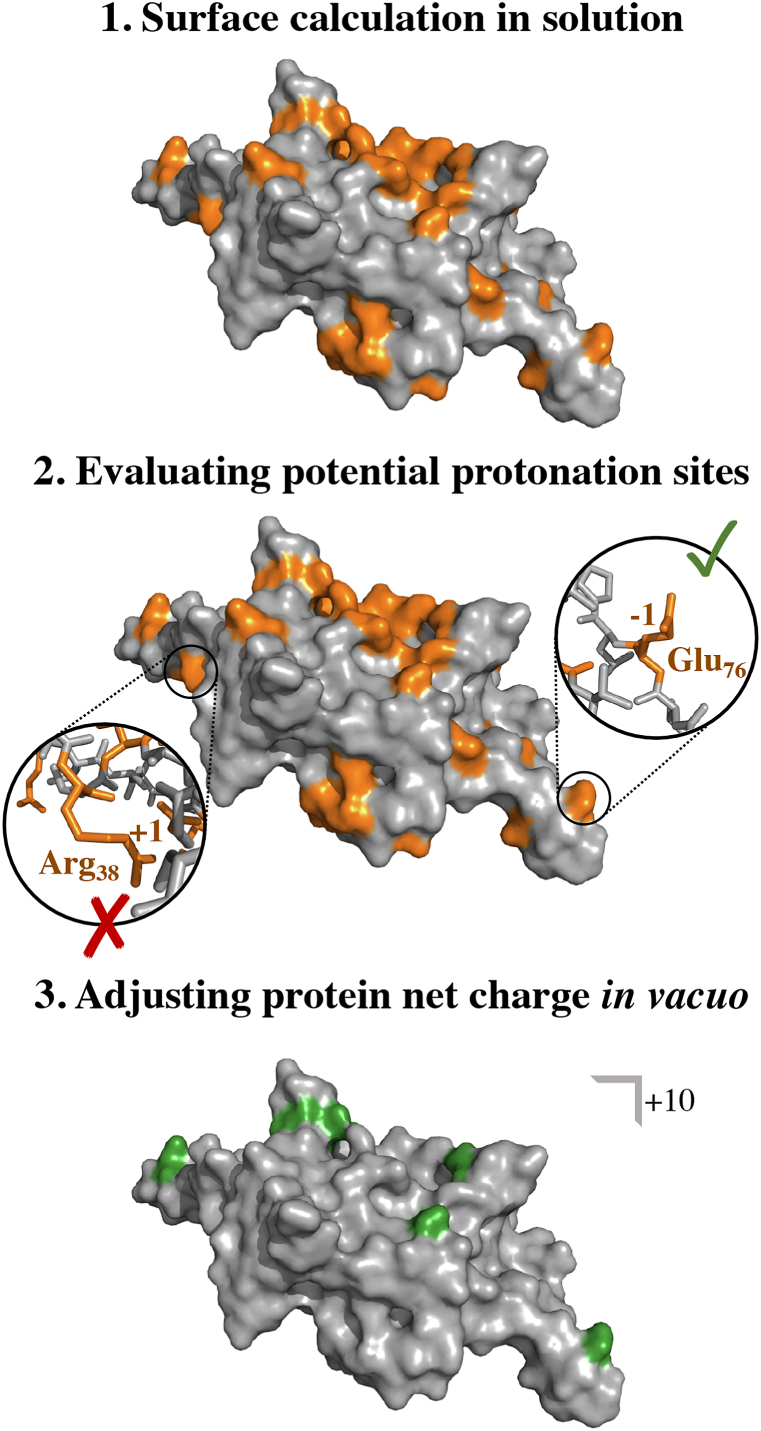
**Adjusting protein protonation for vacuum simulations.** Focus was put on lysine (Lys), arginine (Arg), histidine (His), glutamine (Gln), aspartate (Asp) and glutamate (Glu) as potential protonation sites according to Marchese et al. (2010). At first, the solvent accessible surface area of the selected residues was calculated. Afterwards, the potential protonation sites were evaluated according to their gas-phase basicity, their surface area in solution, and their location within the protein chain, where candidates were ruled out, if a protonation wasn't possible due to Coulomb repulsion. At last, the net charge of the proteins were adjusted to reflect the vacuum charge state reported in literature, +10 *e* (Knapman et al., 2010).

For the vacuum simulations, the Gromacs package version and force field used were kept the same as during solution MD simulations, Gromacs 4.5.7 with the OPLS-AA force field. At first, we assigned the net-charge of the investigated dimers according to published experimental data (Knapman et al., 2010). Consequently, the total charge for the bMS2 dimers was set to +10 *e* (Knapman et al., 2010). To estimate which residues should be protonated, we followed the subsequent procedure (Fig. 2). Focus was put on six amino acids: lysine (Lys), arginine (Arg), histidine (His), glutamine (Gln), aspartate (Asp) and glutamate (Glu). According to Marchese et al. (2010), these residues are most likely to be protonated *in vacuo* in respect to their gas-phase basicity (GPB). Hence, protonation states were exclusively assigned for these six amino acids. In order to pinpoint those residues located on the protein surface, and as such have the highest probability to be protonated in a conventional experimental setup, we calculated the solvent accessible surface area (SASA) per residue using the Gromacs software package. Here, a threshold of 50 ​Å^2^ was applied to disregard residues of smaller area (thus less accessible for exess protons) and limit the number of potential protonation sites. Results of the SASA calculations were sorted according to their GPB (Marchese et al., 2010). From the selection, additional protonation states were assigned until the desired total charge was reached. Candidates were skipped if they already had a positively charged residue as neighbour. As we investigate viral capsid dimers, which as such comprise two identical protein chains, the charges were assigned identically for the two chains. In general, in this study presented averaged data at specific time points are given with the standard deviation, whereas results of averages over time are presented with the standard error.

After assigning the charges, the structures were relaxed *in vacuo* by performing a steepest descent energy minimization followed by a short MD simulation. The temperature was adjusted to 300 ​K with a time step of 0.5 fs over 10 ps using a Berendsen thermostat at a coupling constant of *τ* equal to 0.1 ps (Berendsen et al., 1984). All bonds were constrained using the LINCS algorithm (Hess et al., 1997). In order to mimic ideal vacuum, no periodic boundary conditions nor pressure coupling were applied, and non-bonded interactions were calculated without cut-offs. In subsequent production simulations, protein dynamics were observed over 500 ns at an integration step of 2 fs, employing the Leap-Frog algorithm for integrating Newton's equations of motion (Van Gunsteren and Berendsen, 1988). As such, the C/C and A/B bMS2 dimers were simulated over 200 independent vacuum simulations, resulting in an aggregated simulation time of 100 μs for each system. Details for data analysis are described in more detail in the SI, *Data analysis protocol*.

### 2.3 Rehydration MD simulations and evaluating solution structure recovery

To investigate the behaviour of the here interrogated capsid proteins when taken from vacuum back into solution, the final vacuum simulation structures were extracted and rehydrated in water. Here, the steps taken and parameters applied were exactly the same as provided in the protocol for the initial bulk solution simulations, with only the simulation length being set to 500 ns instead. Consequently, for the rehydration, a total of 200 simulations of 500 ns each were accumulated. All rehydration trajectories were concatenated into a single trajectory for the root mean square fluctuation (RMSF) calculations. Similar to the analysis of the RMSF for the vacuum data, the average structure of all trajectories provided the reference file for the fluctuations calculations. Further data analysis details can be found in the SI, *Data analysis protocol*.

As a result of the hydrophobic properties of the vacuum, exposed proteins will undergo a certain compaction of their respective structure (Rolland et al., 2022). To estimate the extent of said compaction, and how it might be recovered upon resolvation, we calculated the average collision cross-section (CCS) for all vacuum and rehydration simulations using the IMPACT software (Marklund et al., 2015). As reference, the CCS of the initial bulk structures, which were extracted as starting structures for the vacuum simulations, was calculated and averaged. The CCS data and solution recovery analysis was further complemented by the computation of the total surface area and volume of the proteins during the vacuum and rehydration simulations, respectively. Ultimately, to assess the potential recovery of the solution conformation through vacuum-derived structure rehydration, the contacts among residues of the protein chains were calculated and compared to the existing contacts in the original solution structures.

## 3 Results & discussion

### 3.1 Initial solution simulations

Prior to investigating the dynamics of the bMS2 dimers in vacuum and during rehydration, thorough solution simulations of both A/B and C/C bMS2 dimers were carried out to provide starting structures for the vacuum simulations. The dimers were allowed to evolve in solution over 750 ns in ten replicas. The averaged RMSD data of the solution simulations, presented in Fig. S1 in the SI, indicates a slight increase for both dimers until 650 ns of simulation time, after which the dimers seem to reach a plateau at a RMSD value of 2.5 ​Å. Comparing both averaged RMSD graphs in Fig. S1 with each other, the symmetric C/C dimer shows on average an overall higher deviation to the intital protein conformation than the asymmetric A/B dimer. Towards later stages of the simulations, around 500 ns, the RMSD increase lessens, with both graphs levelling out at 2.5 ​Å after approximately 650 ns. Of the last 160 ns of each individual replica simulation, a total of 20 frames taken each 4 ns were extracted, providing starting structures for extensive gas-phase simulations.

### 3.2 Adjusting vacuum protonation

Ahead of taking the extracted solution frames into vacuum, the protonation state of the proteins was adjusted to +10 *e* to reflect the charge state published for mass spectrometry experiments (Knapman et al., 2010). In order to do so, the average SASA of the dimers was calculated, depicted in Fig. S2 of the SI, to indicate which surface amino acids are more exposed to the solvent, and thus are more likely to be protonated. Due to their highly mobile nature *in vacuo*, protons are not fixed to a specific position, but rather are able to migrate along the protein structure (Boyd and Somogyi, 2010; Dongré et al., 1996). This consequently can end up in varying and competing charge locations on the protein, and thus might affect the protein conformations and dynamics. In theory it also allows for multiple low-energy charge configurations with similar energy. The available data is limited, but it seems that there is often one or a few dominating configurations, and that their differences have little effect on the gas-phase structures (Abramsson et al., 2021; Rolland et al., 2022). Whilst proton migration models for MD simulations are subject to ongoing research (Li et al., 2017; Konermann, 2017; Konermann et al., 2021), we followed the aforementioned protocol of assessing and assigning the protonation sites based on SASA calculations and the reported GPB values while taking coulombic repulsion into account, allowing a straightforward approach to mimic protein protonation during ESI, that draws on knowledge from computations in the literature (Schnier et al., 1995; Marchese et al., 2010; Abramsson et al., 2021). The results of protonating the C/C and A/B dimer are presented in Table 1.

**Table 1:** **Adjusted amino acids protonation for the bMS2 dimers *in vacuo*.** Amino acids Asp_11_, Asp_17_, Glu_76_ and Glu_102_ were found to be most likely to protonate transferring from solution to the gas phase. Consequently, their negative charge was neutralized, resulting in a total charge state of +10 *e* for both bMS2 dimers.

**Residue**	**average SASA**	**Charge State**	
	[Å^2^]	*Solution*	*Vacuum*
Asp_11_	87.63 (±4.50)	−1	0
Asp_17_	80.16 (±2.71)	−1	0
Glu_76_	115.58 (±3.97)	−1	0
Glu_102_	76.39 (±1.04)	−1	0

Four amino acids were found to be most likely protonated: Asp_11_, Asp_17_, Glu_76_ and Glu_102_. According to the residue-based SASA calculations, those amino acids obtain on average a solvent-exposed surface area of 87.63 (±4.50) Å^2^, 80.16 (±2.71) Å^2^, 115.58 (±3.97) Å^2^ and 76.39 (±1.04) Å^2^, respectively. Other amino acids of a similar or bigger surface area were eliminated due to their location next to a positive charged residue, because of the more favourable coulombic interaction with the deprotonated form. Both bMS2 dimers were treated equally, meaning the four evaluated residues were protonated in all protein chains from a charge state of −1 to 0. In solution, the total charge of each bMS2 dimer was +2 *e*, resulting therefore in a total charge of +10 *e* of the proteins *in vacuo*.

### 3.3 Root-mean square deviation

After assigning the charges of the A/B and C/C systems, a total of 200 simulations – based on 20 extracted frames per parent bulk trajectory – were started, each running for 500 ns. At first, we calculated the RMSD in order to estimate the behaviour of the vacuum simulations. The average time evolution of the RMSD over all 200 simulations for both bMS2 dimers are depicted in Fig. S3, where one can observe an overall higher mean deviation for the C/C dimer, compared to the A/B dimer. The graph of the A/B dimer protein, starting off at 3.04 (±0.09) Å, shows a continuous, minor increase over 500 ns, reaching a final value at 4.16 (±0.08) Å. The symmetric bMS2 dimer shows a similar trend, from 3.66 (±0.09) Å at the start of the simulations, and ending at 4.90 (±0.07) Å. The general trends of the RMSD graphs suggest that both dimers underwent conformational changes upon vacuum exposure. A potential explanation for the peak RMSD values could be the compaction of proteins induced by the hydrophobic properties in vacuum, as has been reported in the literature for experiments and theoretical studies alike (Rolland and Prell, 2019; Hall et al., 2012). The RMSD for the rehydration simulations was calculated using two different reference structures, namely the final vacuum and bulk solution structure.

Using the final vacuum structure as reference allows us to evaluate the general equilibration and relaxation of the protein structure back in solution, whereas the bulk solution structure as reference permits a first estimation if the proteins find back to their native solution conformation. Average RMSD plots for the rehydration simulations are depicted in Fig. S4. Compared to their individual final vacuum structures as references, the A/B and C/C dimers show a slight increase ending at a final value of 3.63 (±0.24) Å and 3.79 (±0.67) Å, respectively, indicating increasing structural deviations occurring during the 500 ns simulation time. However, in direct comparison with the initial bulk simulation structures, an almost flat RMSD plot is shown for both bMS2 dimers in blue in Fig. S4. Interestingly, whilst the vacuum reference RMSD calculations for both dimers looked very similar, the bulk solution reference RMSD graphs differ considerably. Here, the RMSD of the A/B dimers start from 4.17 (±0.73) Å, and show a final value of 4.21 (±0.91) Å. In comparison, the C/C dimers show an overall increase with a starting value of 5.28 (±0.69) Å, eventually ending at a RMSD of 5.44 (±0.69) Å. These results suggest that, compared to the bulk simulations, the C/C dimers undergo a higher structural deviation during rehydration than the asymmetric conformation. However, the RMSD plots lack detailed information about which parts of the protein structures are deviating, and thus cannot alone justify and evaluate a potential recovery of the initial solution structure. Ideally, if the solution structure would be recovered to a 100% by all 200 replicas, the RMSD plots for the dimers using the bulk solution as reference would show a decreasing trend towards 0 ​Å. Nevertheless, upon rehydration, the dimers seem to equilibrate to the solvent, possibly relax and revert the vacuum-induced compaction, yet further analysis are required to provide insights regarding this.

### 3.4 Root-mean square fluctuation

**Fig. 3:**
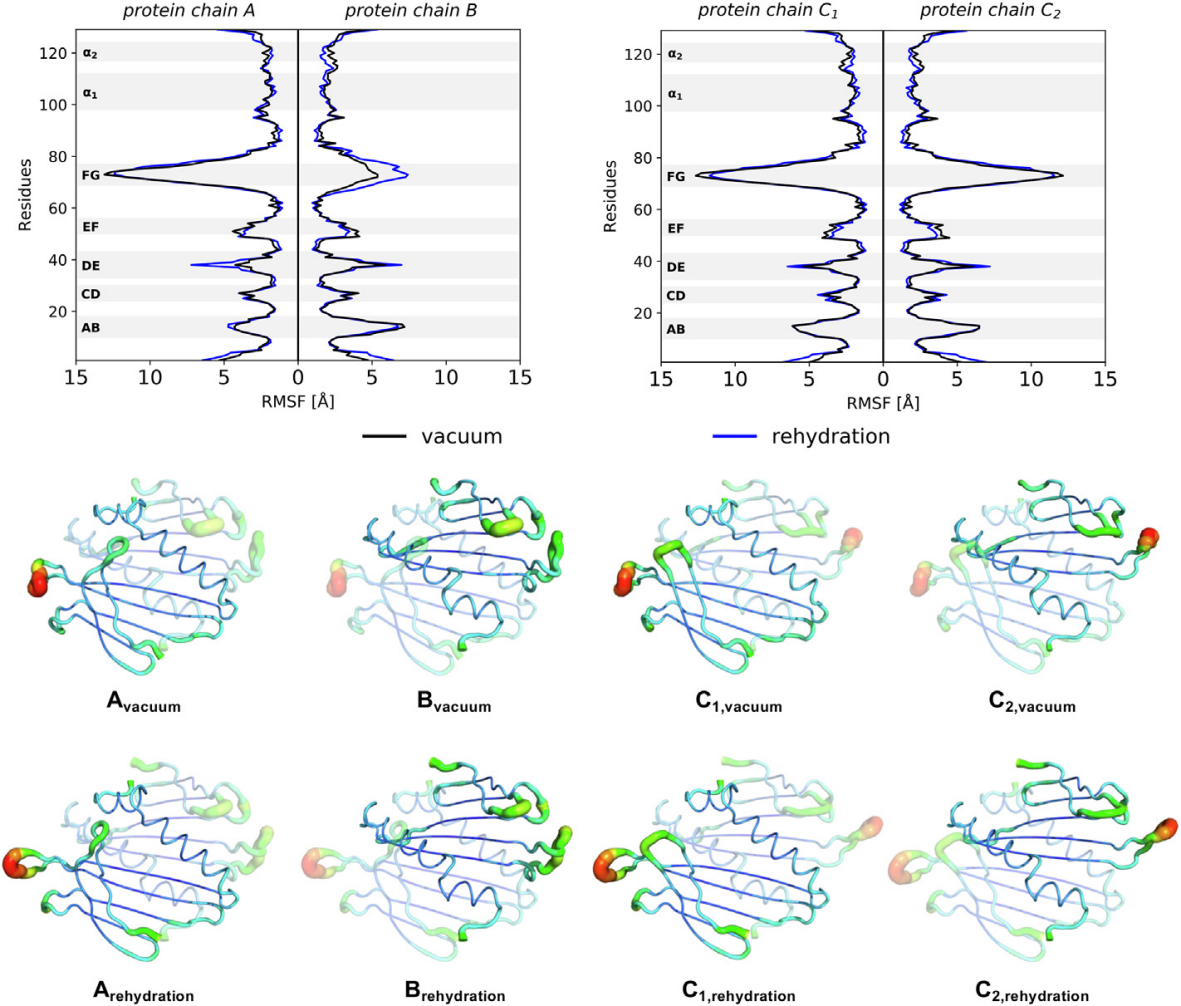
**The RMSF of the individual protein chains.** Comparison of the average RMSF in vacuum and during rehydration, where regions of interest within the protein chains are highlighted. RMSFs are displayed on the crystal structures as B-factor putty representation. Notably, the FG loops differ considerably between the symmetric and asymmetric bMS2 dimers, whilst other *β*-turns possess similar fluctuations. This can further be seen for each individual chain, where the RMSF was plotted onto the protein structures.

The major differences between the two bMS2 dimers are their distinct FG loops, where FG loops in the A and C conformations have defined, anti-parallel *β*-hairpins, whilst FG loops in a B conformation are disordered, collapsed towards the main protein body. Residue-based RMSF data grants information about specific differences in the dynamics of certain residues in the protein chains, pinpointing areas of increased flexibility. The RMSF plots are depicted in Fig. 3.

The trends of the RMSF plots for the symmetric C/C dimer of the individual protein chains, C_1_ and C_2_, are generally alike, both in vacuum and rehydrated, with few occasional differences. Most prominent peaks can be seen between residue 69 and 77. These peaks relate to the FG loop, which in C/C dimers form an extended, defined *β*-hairpin. For the asymmetric A/B bMS2 dimer, one can observe a clear difference between the protein chains in A and B conformation. The A conformation forms, similar to the C conformation, a well-structured FG loop, hence resembling the trend of the RMSF data for the C/C dimers, with a RMSF value in vacuum of 13.09 ​Å, whilst chains C_1_ and C_2_ show peak RMSF values of 12.66 ​Å and 12.14 ​Å, respectively. Residues around the FG loop of chain B of the asymmetric dimer demonstrate an overall smaller fluctuation in vacuum compared to those of the A and C chains, with a peak value of 7.19 ​Å. Upon rehydration, the RMSF values of the FG loop in chain A, C_1_ and C_2_ decreased slightly, to a peak at 12.40 ​Å, 11.72 ​Å and 11.53 ​Å, respectively. Notably, compared to its vacuum fluctuation maximum value, the FG loop of chain B increased during rehydration to 7.41 ​Å. Other peaks in the RMSF graphs are depicting alternative loops and turns in the proteins (Golmohammadi et al., 1993; Perkett et al., 2016). The loop between *β*-strand A and B, around residues 10 to 18, demonstrates a similar fluctuation in that area, both in vacuum and during rehydration for all protein chains. The peak around residues 24 and 30 represents the CD loop, which shows, like the AB loop, an almost identical RMSF trend throughout the vacuum and rehydration simulations. The fluctuation of the DE loop is reflected for the peak around residue 38, displaying for protein chain A a sharp increase between vacuum and rehydration, with the peak RMSF value raising from 4.24 ​Å to 7.21 ​Å, an increase of 70%. Chain B, C_1_ and C_2_ on the other hand present similar values here, both *in vacuo* and rehydration, with values of 5.91 ​Å, 5.43 ​Å and 6.11 ​Å in vacuum, and 6.99 ​Å, 6.47 ​Å and 7.21 ​Å during rehydration, respectively. The EF *β*-strand loop involves residues around 50 and 56, which exhibits higher fluctuation throughout the vacuum simulations for all protein chains compared to its peak RMSF value resolvated. The general structure of both bMS2 dimers possesses two *α*-helices, *α*_1_ and *α*_2_, formed by residues 98 to 111, and 117 to 123, respectively (Golmohammadi et al., 1993). The RMSF data for *α*_1_ and *α*_2_ suggests a similar trend in vacuum and rehydration, with rather slight differences in fluctuations across all protein chains.

In general, the RMSF for the vacuum and rehydration simulations shows similar values for most residues throughout, suggesting that these areas of the protein structures experience analogous fluctuations, which could indicate a recovery of the initial bulk simulation conformation. Overall, the trend for the here presented RMSF calculations resembles the trend published by Perkett et al. (2016), who investigated the mechanisms of the conformational switching in the bMS2 dimers. For that reason, Perkett et al. simulated the A/B and C/C dimers in abscence and in presence of the TR stem loop in solution. However, the study presented here is the first to our knowledge to simulate the bMS2 dimers in vacuum together with subsequent rehydration simulations. The values presented in this study for most *β*-hairpins seem to agree with the values published by Perkett et al.

### 3.5 Collision cross-section

**Fig. 4:**
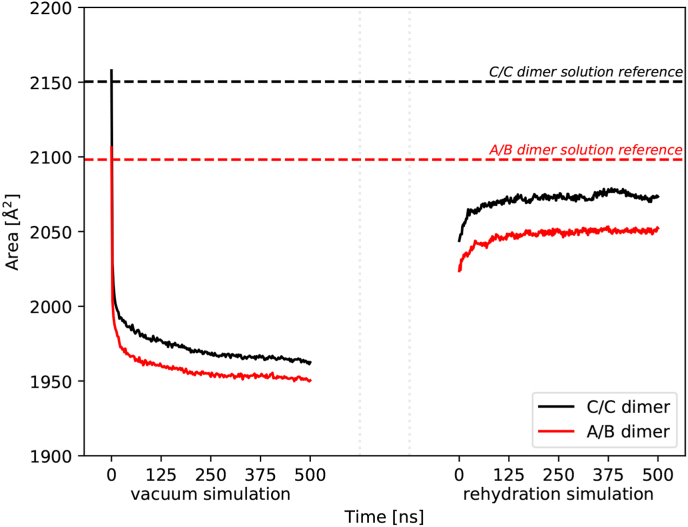
**Comparison of the average CCS data of both bMS2 dimers during the 500 ns of vacuum and rehydration simulations.** The cross-section areas of the proteins were averaged and plotted as dashed baseline in order to estimate the evolution of the CCS over time. Upon vacuum exposure, the proteins compact rapidly within the first few nanoseconds, decreasing their cross-section down to 1950 ​Å^2^. Interestingly, during the rehydration process, the bMS2 dimers show an increase in their CCS data, yet seem to not completely recover the initial solution structures.

In order to further acquire information of the solution structure recovery of both bMS2 dimer variants, we calculated the CCS for each replica, and plotted the results averaged as a function of time. In Fig. 4, the average CCS over the 200 vacuum and rehydration simulations of the A/B and C/C proteins is depicted. Furthermore, the average CCS was calculated for all initial bulk solution structures, allowing a direct evaluation of the recovery process during rehydration. The CCS, being the rotationally averaged area of the two dimensional projection of the protein, is a commonly used metric for gas-phase structural biology (Konijnenberg et al., 2013; Benesch and Ruotolo, 2011). Comparing experimentally determined CCSs with theoretically derived values allows one to probe the conformational heterogeneity and changes in the dynamics of the investigated protein structures. Moreover, calculating the CCS alongside MD simulations provides an additional method complementing theory with experiments (Marklund et al., 2015). Thus, determining the CCS is a suitable means for assessing the solution structure recovery process.

The CCS data shows a decreasing trend over time for both dimers *in vacuo*, starting from a CCS of 2106 (±26) Å^2^ for the asymmetric bMS2 dimer, and 2158 (±25) Å^2^ for the C/C dimer. Compared to the average CCS for the bulk solution data, 2098 (±1) Å^2^ and 2150 (±2) Å^2^, respectively, the initial CCS of the proteins are in agreement with bulk references. The graphs in Fig. 4 demonstrate an overall decreasing evolution of the CCS over time in vacuum. Within the first 2.5 ns, both graphs exhibit a rapid decline, relating to a compaction of the asymmetric dimer by 103 ​Å^2^ (4.9%) to 2004 (±37) Å^2^, and by 129 ​Å^2^ (6%) to 2029 (±42) Å^2^ for the symmetric dimer. From there, the CCS keeps decreasing gradually to a final value at 1950 (±29) and 1963 (±30) Å^2^, for the A/B and C/C dimer, respectively. Knapman et al. report in their study a CCS value for the C/C dimer of 1920 ​Å^2^, based on ion mobility mass spectrometry experiments, a value close to the here presented theoretical CCS for the symmetric bMS2 dimer (Knapman et al., 2010). Furthermore, Knapman et al. conducted theoretical calculations of the CCS as well, and report a value of 1940 (±40) Å^2^. However, in their study, Knapman investigated the asymmetric dimer only with the TR stem loop of the viral genome attached, and thus the reported CCS for the A/B dimer includes in the experiments and the theoretical calculations the cross-section of the stem loop, resulting in a value of around 2000 ​Å^2^. Here, we simulated the A/B dimer without the RNA stem loop, which as a result manifests in a CCS value more similar to the C/C dimer, and thus as well to its reported CCS values.

During rehydration, an increase can be observed for both dimers. Here, the differences between the last CCS in vacuum and the first values during rehydration are due to several pre-production run simulations steps, during which the proteins were already exposed to the solution environment, and thus had time to reequilibrate. Overall, the dimers seem to relax back in solution, which is indicated by the evolution of the CCS over time. The A/B dimer starts from a CCS of 2024 (±41) Å^2^, and evolves over the 500 ns to a final value of 2052 (±40) Å^2^. A similar trend can be seen for the symmetric dimer, indicating an increase of the CCS from 2044 (±46) Å^2^ towards 2073 (±44) Å^2^. Comparatively, both bMS2 dimers demonstrate similar trends during rehydration, yet seem to have maintained their specific conformations, indicated in the offset of the starting values. The reference baselines in Fig. 4 provide the information of the CCS of the initial bulk simulations, and therefore can be used to gauge the recovery process of the solutions structures. As the last 50 ns of the CCS graphs, both in vacuum and during rehydration, demonstrate a relatively linear trend, we used these 50 ns for the solution structure recovery estimations. During the last 50 ns, the dimers compact in vacuum to 1952 (±1) Å^2^ and 1965 (±1) Å^2^, for the A/B and C/C dimers, respectively, implying a total compaction of 6.96% and 8.64%. During rehydration however, the CCS data of the dimers show a difference between reference and the average value of the last 50 ns, 2047 (±5) Å^2^, of 51 ​Å^2^ for the A/B dimer, and 80 ​Å^2^ with an average value of 2070 (±6) Å^2^ for the symmetric C/C dimer. This would indicate a solution conformation recovery of 97.54% and 96.27% for the A/B and C/C dimer, respectively, by the means of the theoretical CCS of the bMS2 dimers. This is further supported by calculations of the total surface area and volume of the proteins, where over the last 50 ns of the rehydration simulations the suface area and volume of both dimers recovered by 100%, and 99.90%, respectively (see 10.13039/100013443SI, *Data analysis protocol*).

### 3.6 Contact maps

**Fig. 5:**
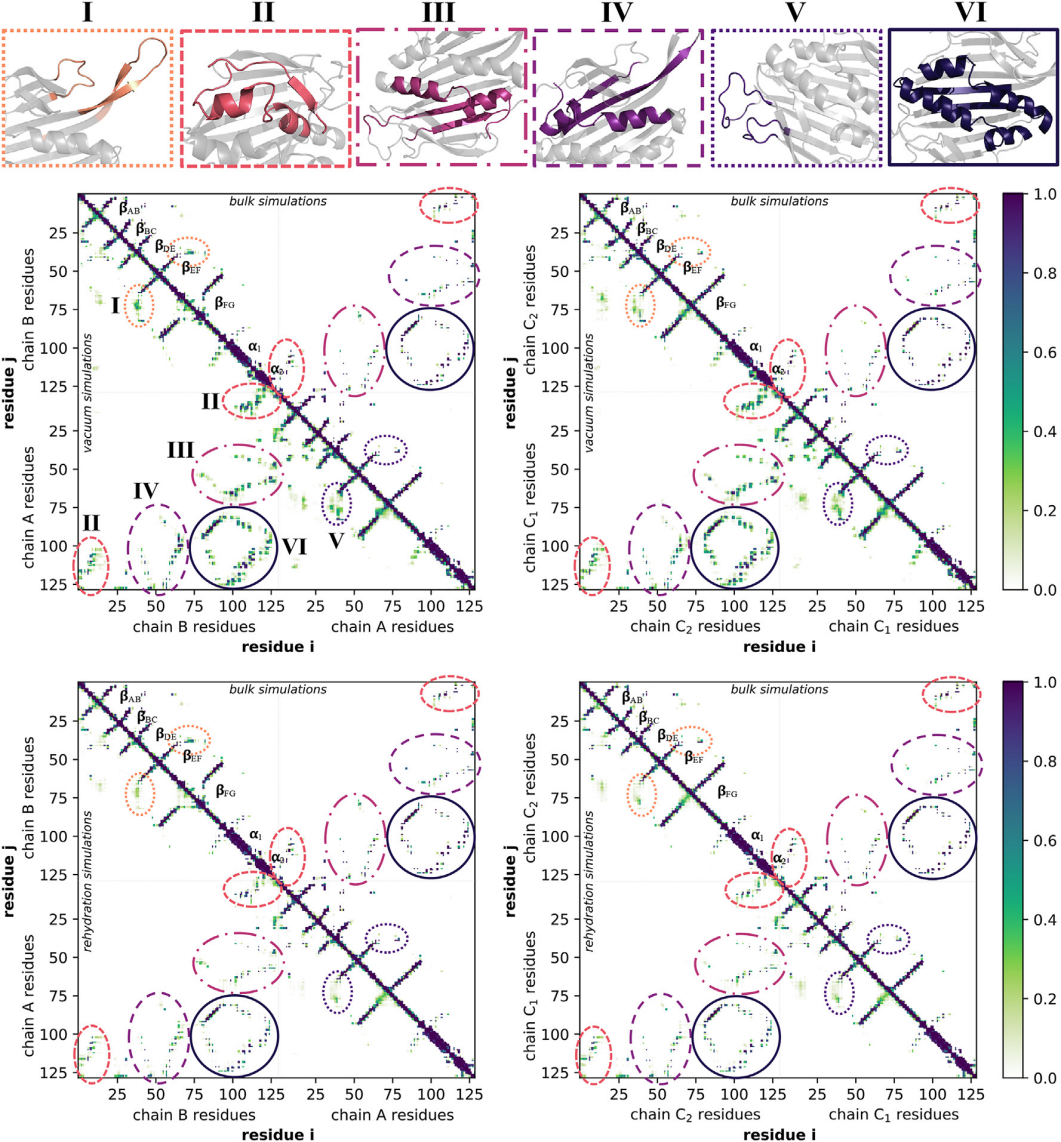
**Contact maps of the last 50 ns in vacuum and during rehydration in comparison with the initial bulk simulation data.** Maps depicted in the upper row reflect the comparison of the vacuum contacts for the A/B and C/C bMS2 dimer (left to right, respectively shown in the lower triangular matrix) versus the contacts present during the initial bulk simulations (upper triangular matrix). Average normalized contacts during the rehydration simulations are illustrated in the lower row, with the data for the A/B and C/C dimers shown in the lower triangular matrix. Contacts were defined as existing, if the two evaluated residues had a distance within 3.5 ​Å of each other, and further plotted to account for the 200 simulated replicas, where a value of 0 on the scale reflects that no contact existed throughout all 200 simulations. The data suggests that upon vacuum exposure, new contacts form in both A/B and C/C dimers, as a result of a compaction of the protein structures due to the hydrophobic environment. Furthermore, during rehydration, most of the contacts seem to revert partially back, resembling those during the bulk. Nevertheless, as can be further seen from the rehydration plots, the bulk structures are not totally recovered, and few vacuum-derived contacts still remain present.

With the CCS, protein area and volume calculations granting the first proof of a general solution structure recovery through gas-phase structure rehydration, those results fail to provide detailed information of the distinct areas of the proteins which ‘remember’ their initial solution conformation, and those areas which yet divert from their bulk solution structure. In order to shed further light into this, we generated contact maps of the bMS2 dimers, both in vacuum and during rehydration, and compare those versus the existing contacts present in the initial solution structure. Supported by the CCS calculations, we focused here on the contacts of the last 50 ns of the vacuum and rehydration simulations. Eventually, the generated contact maps were then plotted versus the bulk solution contact maps, which allows a direct comparison and estimation of the solution recovery process. Here, the bulk solution maps show identical contacts as published in literature (Lalwani Prakash and Gosavi, 2021). The thus merged maps show the vacuum or rehydration contacts in the lower triangular matrix, the bulk solution contacts in the upper matrix. As both bMS2 dimers are sequence-identical, it is necessary to distinguish between the specific monomeric chains in order to pinpoint potential differences and similarities between them. The axes of the contact maps are therefore divided to show the residues for each individual protein chain. The final contact maps are presented in Fig. 5.

Here, contacts between the residues of the DE and FG loop are marked with I and V, for each specific protein chain respectively. As the FG loops are the most prominent conformational differences between the protein chains A, B and C, contacts involving the residues of the FG loops might provide detailed information if the structures maintain their initial conformation. Marked with II are the contacts between the residues of the *β*-strand A and of those in the *α*-helices of the opposite protein chain. Residues of the E and F *β*-strands, which are potentially in contact with those of the *α*-helices, are marked III and IV, separately marked and coloured to account for the differences of the F strand residues of the symmetric and asymmetric conformations, whereas VI in Fig. 5 encloses the contacts between G *β*-strand and *α*-helices residues. In a contact map, *β*-sheets can be observed as perpendicular bands to the diagonal, whilst *α*-helices show bands parallel to it, which are marked accordingly in Fig. 5. Focusing on the residues that make up the secondary structures of the bMS2 dimer therefore further allows the distinct estimation of the solution structure recovery, providing more information of the underlying dynamics and conformational changes.

In general, we observed that new contacts are present during the last 50 ns of the vacuum simulations for both dimers, as a result of the compaction of the protein structure. More in detail, comparing the contacts between the residues of the DE and the FG loop of chain A in vacuum and bulk with each other, it is evident that more contacts have formed in vacuum. With these new contacts having an occupancy between 0.2 and 0.4, this would indicate that rather than a full compaction towards the protein body, the FG loop comes in contact with the residues of the DE loop due to its high fluctuation. Our RMSF calculations support this observation, where the FG loops fluctuates on average in vacuum by 13.09 ​Å. The vacuum contacts between the residues of the DE and FG loops for protein chain C_1_ show the same observation as for chain A, where the newly formed contacts are most likely to be the consequence of the fluctuation of the C_1_ chain during the simulations. Similar contacts of the same residues can further be seen for chains B and C_2_, whereas here the contacts for protein chain B are less likely due to its fluctuation in vacuum, but rather due to its already collapsed conformation, which brings it closer to the residues of the DE loop. This is supported by the already existing contacts between these residues of the initial bulk data for chain B, which are not present for chains A, C_1_ and C_2_. Upon rehydration, the number of contacts between the DE and FG loop decreased, indicating that most of the contacts exist below 20% during all simulations, and thus most likely due to the fluctuations of these residues. Contacts between residues 1 to 12 of the *β*-strand A and the *α*-helices with the C-terminus, more specifically residues 106 to 129, reveal in general shorter distances between the two monomer chains in vacuum. Moreover, this observation holds true for all protein chains, indicating a compaction of the protein structure in that area. The rehydration data demonstrates still existing contacts in contrast with the contacts during the bulk solution simulations, both for the symmetric and asymmetric bMS2 dimers. With occupancies around 0.3, these contacts might be due to the increased fluctuation of the A *β*-strand. Our RMSF calculations imply that the *α*-helices are rather immobile, with termini residues of all chains showing a fluctuation in vacuum and during rehydration of around 5 ​Å, which could explain the observed contacts as *β*-strands are more fragile compared to *α*-helices in the gas phase (Seo et al., 2016). Further protein structure compaction between residues of the E and F *β*-strands and the *α*-helices is indicated by additional intramolecular contacts in vacuum, which are missing in the bulk solution reference. Contacts show here an occupancy between 0.4 up to 0.7, revealing that the compaction around that area is present throughout the majority of the simulations. Here, both bMS2 dimers have similar contact patterns, indicating that both dimers compact equally during the last 50 ns in vacuum. Rehydrating the dimers implies that the major part of the compaction was reversed, as shown by fewer interactions present for the contacts between E, F *β*-strands and the *α*-helices. However, compared versus the bulk solution contacts, the data reveals a small number of interactions still present over the last 50 ns of the rehydration simulations. Residues 84 to 125 make up the G *β*-strand and *α*-helices of the bMS2 dimers, and thus allow the observation of the dynamics of the protein core. Consequently, *in vacuo* protein compaction would be reflected by a significant increase between these residues. As demonstrated in the vacuum contact maps, in comparison with the solution reference, additional contacts formed in vacuum, with the majority showing an occupancy above 0.5. Interactions between the protein chains A and B demonstrate a similar pattern as shown between C_1_ and C_2_. However, a minor difference can be seen, as contacts between residues 84 to 86 of chain B and *α*_1_-residues of chain A are missing, potentially due to the unstructured conformation of the FG loop of protein chain B, preventing the formation of intramolecular contacts here. Nonetheless, the observed compaction seems to be largely identical between A/B and C/C dimers.

The rehydration results imply, as observed for other areas of the proteins, that the majority of the contacts formed in vacuum reverse, and that the protein core relaxes back in solution. Whilst very few contacts remain, the pattern observed here is nearly identical to the bulk reference, further supporting an almost complete recovery of the initial solution structure, which holds true for both the symmetric and aysmmetric bMS2 dimer, as both dimers suggest an equal recovery. This is further supported by the higher number of average hydrogen bonds present during the analysed 50 ns of the vacuum, approximately 257 for both dimers, compared to the rehydration simulations, with 171 and 168 hydrogen bonds for the A/B and C/C dimer, respectively, as reported in literature (Van der Spoel et al., 2011). Overall, the contact maps presented here reveal that the rehydration of the structures originating from the vacuum simulations resulted in the recovery of the majority of the initial bulk solution conformation. Few contacts are still present during the last 50 ns of the rehydration simulations, which cannot be observed in the bulk reference, most likely due to differences in fluctuations seen for the specific parts of the protein structure.

According to literature, the A/B dimer conformation exists predominately in contact with the viral genome; consequently, the absence of it prompts the formation of the symmetric dimer in solution (Knapman et al., 2010; Stockley et al., 2007). Throughout our simulations, we regularly observe differences between the two simulated dimers, both in vacuum and during rehydration. However, despite the extensive simulations, we did not observe any conversion from the asymmetric towards the symmetric conformation of the bMS2 dimers. Potentially, the energy barrier between the conversion from asymmetric to symmetric bMS2 dimer might be too high in the gas phase, thus preserving the conformations in these states. Upon rehydration, as the structures find their way back to their respective initial conformation, the switch from A/B to C/C might happen on time scales longer than have been explored in this study, and thus could potentially be observed by enhancing the simulation time scale.

## 4 Conclusion

Proteins differ in how easily they can be transferred to the gas phase without loss of structure, but even when their overall architectures remains unaltered, vacuum exposure is known to affect the structures to some degree also under the most gentle conditions. Probing and estimating these effects represents an immense challenge experimentally, often lying outside of the view of the techniques used. In contrast, MD simulations are able to trace these specific structural differences on an atomistic level, and thus provide a potential means to refine experimental structures obtained from the gas phase and obtain native-like conformations.

Here, we present extensive MD simulations of the two existing conformations of the bMS2 dimer with the aim of probing their stability during vacuum exposure, and moreover the ability of the structures to recover their initial solution structure upon rehydration. Our simulations suggest that the majority of the investigated protein structures find their way back and ‘remember’ their initial solution conformation prior to vacuum exposure, as most of the intramolecular contacts are restored during rehydration. At the same time, we do not observe any interconversion between the two dimer states in the process, suggesting that the conformations occupy separate basins in the folding landscape of the protein also in the gas phase, where barriers are expected to be higher than in solution, so that the local conformational exploration during the resolvation simulations find the original state rather than the global free energy minimum, which in this case would be the C/C dimer conformation. This consequently presents rehydration simulations as a promising supplementary method to further enhance gas-phase protein structure determination, enabling more biologically relevant structures to be obtained from such experiments.

Our investigation pertains to native ion mobility mass spectrometry, where experimentally derived CCSs enable modelling of the protein structures as they appeared in the gas phase, albeit at a low spatial resolution (Marklund and Benesch, 2019). There is a discrepancy between modelled and experimental CCSs that in part can be explained by slight adjustments of proteins’ structures to the vacuum conditions. As such our results tie in with earlier computational studies of mainly monomeric proteins under ESI-like conditions (Patriksson et al., 2007; Marklund et al., 2009; Meyer et al., 2009; Rolland and Prell, 2019; Rolland et al., 2022). In contrast to most other MD studies of gas-phase proteins, we have seen how structural changes to the proteins in the gas phase not only involves general compaction and side-chain collapse on the surface, but deformation of the *β*-hairpin that is distinctive for the two conformers. The absence of interconversion between A/B and C/C dimers are in line with earlier observations from gas-phase simulations of lysozyme, where trajectories starting from very closely related starting structures display lower levels of structural dynamics within each trajectory than the difference between trajectories, reflecting a rugged energy landscape in the gas phase, with structurally related basins with barriers between them that prevent interconversion (Mandl et al., 2020). It is intriguing to see that such separation carries over to a subsequent rehydration step, even in absence of the A/B-stabilizing RNA.

Recent experiments with mass-selected proteins in soft landing experiments by Westphall et al. (2022) show how long exposure to vacuum – on the order of 10 ​min – can lead to deformation of the tertiary or quartenary structure. Deposition on a glycerol-coated surface on the other hand, which acts to reverse the dehydration, was successful in preventing structural loss. Esser et al. (2022a) however achieved structurally intact protein complexes in a similar approach without any such coating, but where the landed particles retained some of the most closely bound water. Neither study could produce images that allowed 3D-reconstructions on a resolution that allowed inspection of the structures on the level of detail that would reveal the changes observed on our investigation. Soft landing for cryo-EM is still under development, and ongoing efforts to reduce or eliminate factors that are destructive for the structure are being made (Esser et al., 2022b). As the field advances, opportunities will likely present themselves to bridge our computational investigations with experimental observation. To connect with our results in the time domain however, we need either substantially longer simulations, or other types of experiments that take place on sub-μs time scales, such as future single particle imaging at high resolution. The apparent kinetic stability observed in gas-phase simulations and indicated by both soft landing and native mass spectrometry suggest however that the rearrangements we see in the proteins upon vacuum exposure are indeed also informing about what happens on longer time scales.

## CRediT authorship contribution statement

**Maxim N. Brodmerkel:** Conceptualization, Methodology, Formal analysis, Investigation, Validation, Writing - Original Draft, Writing - Review & Editing, Visualization. **Emiliano De Santis:** Methodology, Software, Writing - Review & Editing. **Charlotte Uetrecht:** Conceptualization, Writing - Review & Editing, Supervision, Funding acquisition. **Carl Caleman:** Conceptualization, Writing - Review & Editing, Supervision, Funding acquisition. **Erik G. Marklund:** Conceptualization, Resources, Writing - Review & Editing, Supervision, Funding acquisition.

## Declaration of competing interest

The authors declare that they have no known competing financial interests or personal relationships that could have appeared to influence the work reported in this paper.

## Data Availability

Data will be made available on request.
