# Supplementary material for: Stability and conformational memory of electrosprayed and rehydrated bacteriophage MS2 virus coat proteins
Source: Curr Res Struct Biol. 2022 Nov 4;4:338–48. doi: 10.1016/j.crstbi.2022.10.001 (PMC9685359; doi:10.1016/j.crstbi.2022.10.001)
Supplement: Multimedia component 1 [file mmc1.pdf]

Supplementary Information: Stability and conformational memory of electrosprayed and rehydrated bacteriophage MS2 virus coat proteins

Maxim N. Brodmerkel[1], Emiliano De Santis[1,2], Charlotte Uetrecht[3,4,5], Carl Caleman[2,6], and Erik G. Marklund*[1]

[1]Department of Chemistry – BMC, Uppsala University, Box 576, 751 23 Uppsala, Sweden
[2]Department of Physics and Astronomy, Uppsala University, Box 516, 751 20 Uppsala, Sweden
[3]Leibniz Institute of Virology (LIV), 20251 Hamburg, Germany
[4]Centre for Structural Systems Biology (CSSB), Deutsches Elektronen-Synchrotron, DESY, Notkestrasse 85, 22607, Hamburg, Germany
[5]School of Life Sciences, University of Siegen, Siegen, Germany
[6]Center for Free-Electron Laser Science, DESY, Notkestrasse 85, 22607 Hamburg, Germany

November 20, 2022

1 Supplementary Information

1.1 Data analysis details

1.1.1 Root-mean-square deviation and fluctuation

The vacuum data, and with it the behaviour of the systems *in vacuo*, was at first analysed by calculating the RMSD and RMSF. Here, the first frame of each individual trajectory was used as reference structure for RMSD calculations. For the RMSF calculations, at first, all trajectories were concatenated into a single trajectory, of which the average structure was calculated. This average structure was then further taken as reference to calculate the RMSF of the protein residues on the combined trajectory.

Similar to the vacuum simulations, at first, RMSD and RMSF calculations were conducted to estimate the behaviour of the systems. Here, the specific RMSDs of the rehydration simulations were calculated based on two specific reference structures. The vacuum structure, starting structure of the rehydration simulations, as reference allowed for the inspection of the general dynamics of the bMS2 dimers and the equilibration to the solvent. On the other hand, providing the initial bulk solution simulation structures as references gave first insights on a potential recovery of the original solution structures of the dimers.

1.1.2 Total surface area and volume

Recovering the solution conformation from a vacuum-derived structure is inevitably linked to a relaxation of the protein in solution, and with it a reversal of the vacuum compaction. Consequently, a successful solution structure recovery would be indicated by an increase of the protein surface area and volume towards their initial solution values, which were calculated to complement our theoretical CCS calculations further. Trends of the protein surface area and volume during the vacuum and rehydration simulations are depicted in figure S5 of the SI.

The total protein surface area of the A/B dimers extracted from the initial bulk simulation was on average 14994 Å^2, for the C/C dimers 15021 Å^2 - both values used as solution reference. The initial values of the vacuum simulations of 15087 Å^2 and 15110 Å^2 for the asymmetric and symmetric dimers, respectively, are larger than the solution reference, likely due to the same reasons as explained for the CCS. The protein area of the A/B dimer decreases by 382 Å^2 (2.5 %) over the first 2.5 ns of simulation, the C/C dimer over the same time span by 377 Å^2 (2.5 %). Over the rest of the simulation time, the dimer area for both proteins decrease further, to a final value of 14550 Å^2 for the A/B dimer, and 14555 Å^2 for the C/C dimer. Estimating the compaction over the last 50 ns, the total surface area decreased to an average value of 14557 ($\pm$ 2) Å^2 for the A/B dimer, and 14580 ($\pm$ 2) Å^2 for the C/C dimer, indicating a total reduction of the protein surface area over 500 ns in vacuum by 3.5 % for both bMS2 dimers. Rehydrating the vacuum structures allowed the total area to relax and expand, increasing from 14980 Å^2 to 15023 Å^2 for the asymmetric dimer, and for the C/C dimer from 15050 Å^2 towards a surface are of 15064 Å^2. Here, over the last 50 ns, the average surface area of the dimers demonstrate values of 15014 ($\pm$ 2) Å^2 and 15061 ($\pm$ 2) Å^2, for A/B and C/C, respectively. This indicates a recovery of the total surface area of the protein of 100% for both dimers, and with it interestingly a slight increase.

Similar results were obtained from the protein volume calculations, which demonstrate in vacuum initial values of 24878 Å^3 and 24873 Å^3 for the A/B and C/C dimers. The first 2.5 ns reveal a decrease of the volume for the asymmetric dimers by 1 % towards 24637 Å^3, whereas the symmetric dimer volume decreases to 24659 Å^3 by 0.9 %. At the end of the vacuum simulations, the dimers obtained a volume of 24609 Å^3 and 24618 Å^3, respectively for the A/B and C/C dimers. Here, the last 50 ns revealed a compaction during vacuum to an average protein volume of the A/B dimer of 24603 ($\pm$ 3) Å^3, and 24621 ($\pm$ 2) Å^3 , thus implying a vacuum compaction by 1.1 % and 1 %. During rehydration, the volume reveals an evolution to 24721 Å^3 of the A/B dimers, and towards 24745 Å^3 for the C/C dimer. Estimating the solution structure recovery by the means of protein volume, the last 50 ns show on average a value of 24722 ($\pm$ 2) Å^3 and 24739 ($\pm$ 2) Å^3, respectively for the asymmetric and symmetric bMS2 dimers. In other words, the A/B dimers recover the initial solution volume to 99.9 % compared to the reference protein volume of 24741 Å^3, and by 99.9 % for the C/C dimers, with a respective reference volume of 24749 Å^3.

1.1.3 Contact maps generation

At first, the distances between all atoms were calculated using the MDAnalysis python package (Gowers et al., 2016; Michaud-Agrawal et al., 2011), conducted for the initial bulk solution structures, and the last 50 ns of the respective vacuum and rehydration data sets. Herein, pairs of residues were screened by applying a cut-off

of 3.5 Å, where a contact between the residues *i* and *j* was defined as existing, if the distance between at least one atom of residue *i* and residue *j* was found within the cut-off. Consequently, no contact was assigned if the distance between two residues was outside 3.5 Å. The final contact list of the vacuum and rehydration data was then respectively mapped versus the contacts within the initial solutions structures. Here, the contact maps were normalized to show each contact adopting a value within the scale of 0 to 1. This allows to further account for the 200 individual replicas, with contacts of a value of 1 meaning that during all 200 simulations that specific pair of residues was consistently in contact. Ergo, a value of 0 demonstrates that no contact was present throughout the simulations.

1.2 Additional Figures

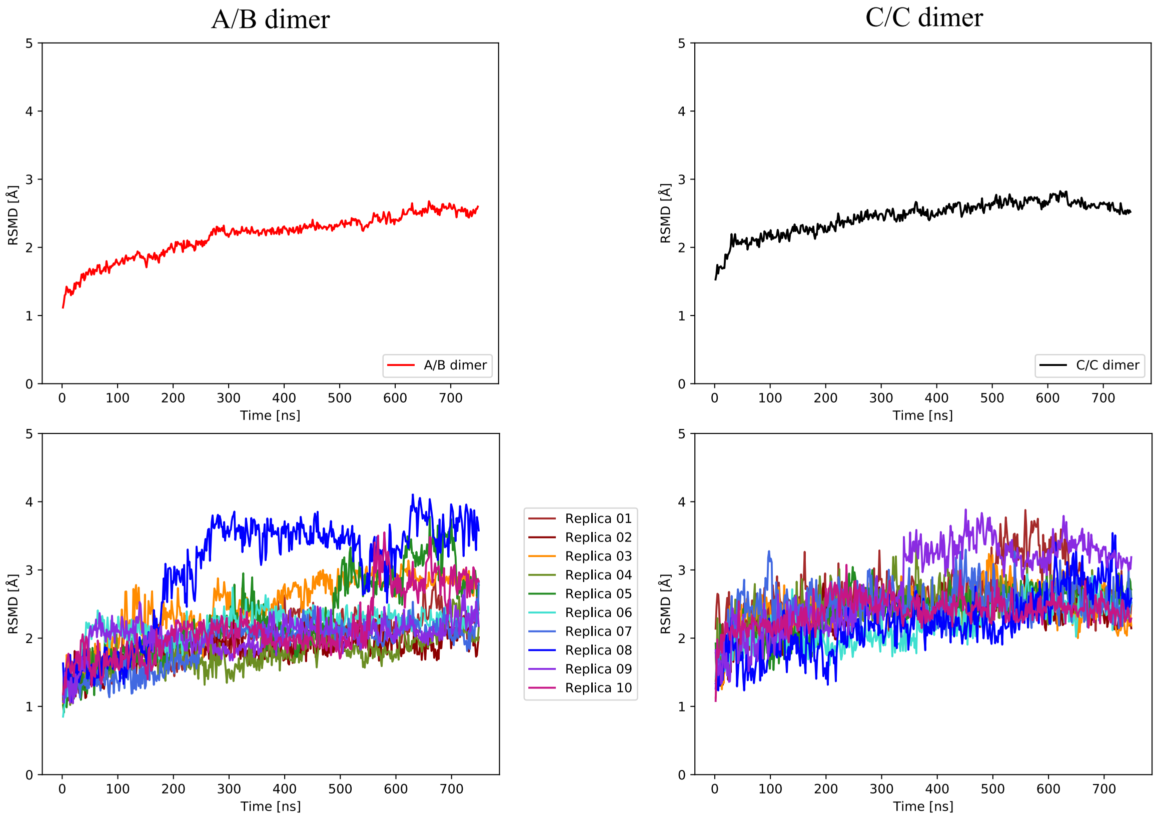

Figure S1: **RMSD of each bulk solution simulation.** The averaged evolutions of the RMSD for both bMS2 dimers indicate the replicas, shown below, reaching a plateau around 2.5 Å after 650 ns of simulation. From the last 160 ns, for each replica, a total of 20 structures every 4 ns was extracted as starting structures for the vacuum simulations.

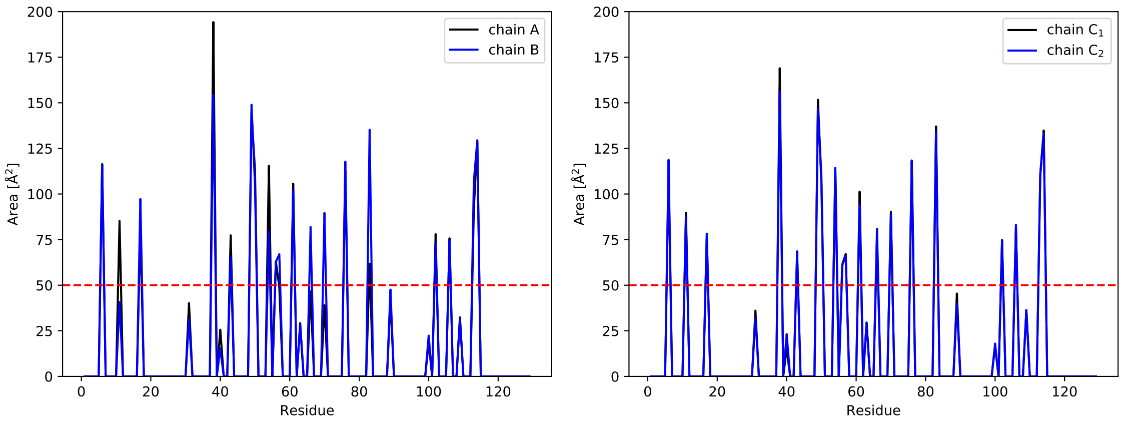

Figure S2: **SASA of both bMS2 dimers.** The SASA was calculated in order to pinpoint potential protonation sites of lysine, arginine, histidine, glutamine, aspartate or glutamate, which were found to have the highest GPB according to Marchese *et al.* (Marchese et al., 2010). The red line indicates the applied threshold of 50 Å^2, which excludes amino acids with a smaller surface area.

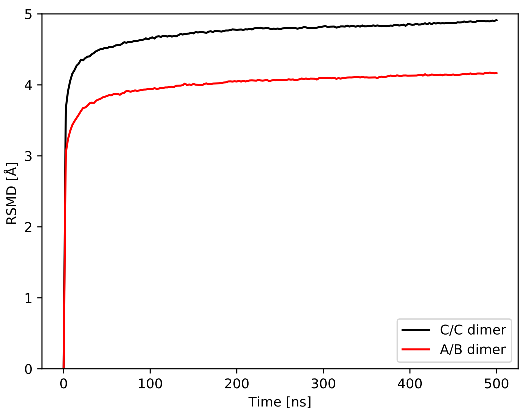

Figure S3: **Averaged RMSD of both bMS2 dimers in vacuum.** The averaged time-evolved RMSDs of the A/B and C/C dimers show overall an increase throughout the simulations, suggesting considerable conformational changes *in vacuo*.

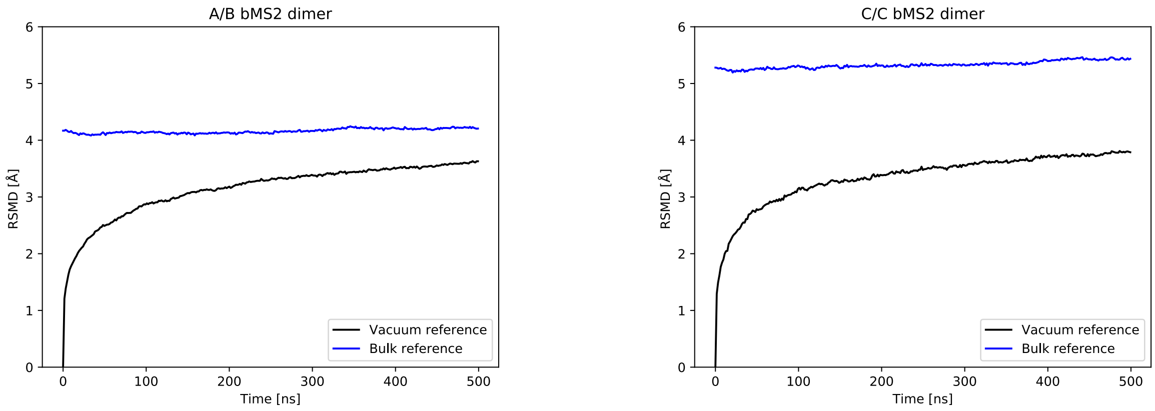

Figure S4: **Averaged RMSD of both bMS2 dimers during rehydration.** Rehydration-RMSD calculations were conducted using two different structures as references. Using the final vacuum structures as reference allows a general estimation of the underlying dynamics upon rehydration. Using the original bulk structures however gives a first estimation of a structural recovery of the initial solution conformation of the individual proteins.

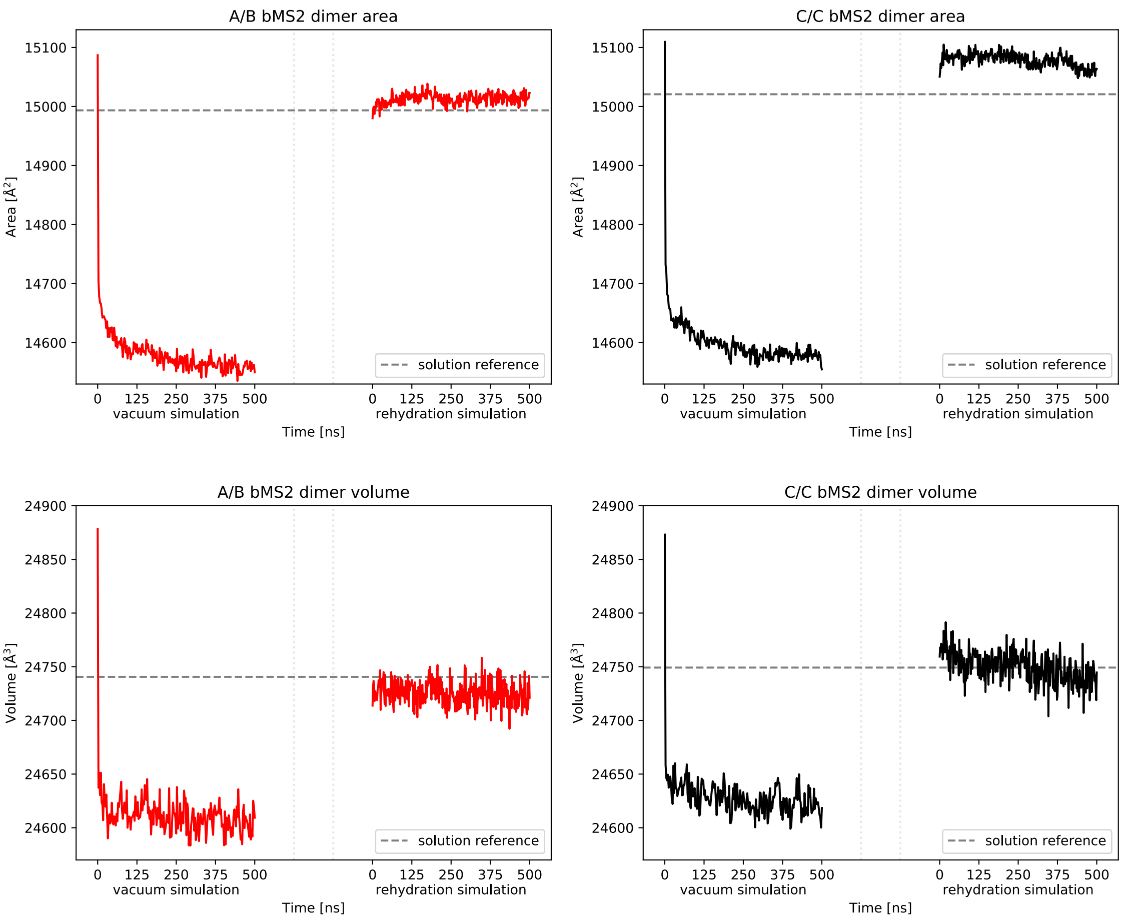

Figure S5: **Protein area and volume in vacuum versus during rehydration.** Whilst in vacuum both dimers show a decrease of both total area and volume, the rehydration data suggest an approximation towards the specific bulk solution reference (dashed line).

References

Gowers, R.J., Linke, M., Barnoud, J., Reddy, T.J.E., Melo, M.N., Seyler, S.L., Domanski, J., Dotson, D.L., Buchoux, S., Kenney, I.M., Beckstein, O., 2016. MDAnalysis: a python package for the rapid analysis of molecular dynamics simulations. Proceedings of the 15th Python in Science Conference , 98–105doi:10.25080/Majora-629e541a-00e.

Marchese, R., Grandori, R., Carloni, P., Raugei, S., 2010. On the zwitterionic nature of gas-phase peptides and protein ions. PLoS Comput. Biol. 6, e1000775. doi:10.1371/journal.pcbi.1000775.

Michaud-Agrawal, N., Denning, E.J., Woolf, T.B., Beckstein, O., 2011. MDAnalysis: a toolkit for the analysis of molecular dynamics simulations. J. Comput. Chem. 32, 2319–2327. doi:10.1002/jcc.21787.
